# Simplified platform for mosaic *in vivo* analysis of cellular maturation in the developing heart

**DOI:** 10.1038/s41598-019-47009-7

**Published:** 2019-07-24

**Authors:** Julie Goudy, Trevor Henley, Hernán G. Méndez, Michael Bressan

**Affiliations:** 1 Department of Cell Biology and Physiology, University of North Carolina at Chapel Hill, Chapel Hill, USA; 2 McAllister Heart Institute, University of North Carolina at Chapel Hill, Chapel Hill, USA

**Keywords:** Transgenic organisms, Genetic transduction

## Abstract

Cardiac cells develop within an elaborate electro-mechanical syncytium that continuously generates and reacts to biophysical force. The complexity of the cellular interactions, hemodynamic stresses, and electrical circuitry within the forming heart present significant challenges for mechanistic research into the cellular dynamics of cardiomyocyte maturation. Simply stated, it is prohibitively difficult to replicate the native electro-mechanical cardiac microenvironment in tissue culture systems favorable to high-resolution cellular/subcellular analysis, and current transgenic models of higher vertebrate heart development are limited in their ability to manipulate and assay the behavior of individual cells. As such, cardiac research currently lacks a simple experimental platform for real-time evaluation of cellular function under conditions that replicate native development. Here we report the design and validation of a rapid, low-cost system for stable *in vivo* somatic transgenesis that allows for individual cells to be genetically manipulated, tracked, and examined at subcellular resolution within the forming four-chambered heart. This experimental platform has several advantages over current technologies, chief among these being that mosaic cellular perturbations can be conducted without globally altering cardiac function. Consequently, direct analysis of cellular behavior can be interrogated in the absence of the organ level adaptions that often confound data interpretation in germline transgenic model organisms.

## Introduction

The developing heart presents unique challenges for biomedical investigation. During cardiac morphogenesis, juvenile cardiomyocytes undergo cellular diversification, cytoarchitectural specialization, and functional integration as the heart loops, septates, and coalesces into a highly coordinated muscular pump. Importantly, each of these processes occur under the influence of systemic increases in hemodynamic force and rapid cyclical changes in biophysical stretch and strain^1–6^. As a result, it is difficult to model cardiac development in experimental systems that do not replicate the native stresses present in the embryonic heart.

While increasingly sophisticated germline genetic models are continuously being developed to conduct mechanistic studies into heart formation, these systems have several drawbacks. In particular, germline genetic manipulations frequently target large populations of cells in the heart altering function at both the cellular and organ level. This can greatly complicate interpretation, making it difficult to discriminate between direct consequences of a genetic perturbation and indirect effects that arise secondary to altered cardiac electromechanical activity^7,8^. Furthermore, the time and cost associated with generating and maintaining transgenic lines can limit the speed and number of factors that can be interrogated experimentally, which serves as a significant bottleneck in attempting to assign biological function to gene and protein networks that are now routinely being identified by modern high throughput sequencing platforms. Cell culture systems can be used to supplement current *in vivo* models, however, most standard culture conditions lack the three-dimensional architecture and dynamic biophysical interactions present within the developing heart. Consequently, cardiac research would greatly benefit from an experimental system that could bridge the current gap between germline transgenics and *in vitro* conditions.

From a design perspective, an ideal experimental platform for overcoming current obstacles in developmental cardiac research should possess several features: the system would replicate four-chambered heart development with high fidelity, cells and tissue would be highly accessible throughout the experimental manipulation, genetic perturbations would be rapid and low cost, physiological behavior would be easily assayable, hearts would be amenability to high-resolution imaging, and sufficient cells would be generated to allow for downstream transcriptional and proteomic analysis. Prioritizing these criteria has led us to focus on the chick embryo as a potential foundational model system on which to build a simple *in vivo* experimental system that allows for a novel form of developmental cardiac bioengineering.

Although it has a long history as a classical model of four-chambered heart development, the chick has not been utilized as a true genetic model system. This is, in part, due to the fact that genetic modifications through the germline are difficult and the resulting transgenic flocks are not easy to maintain in a laboratory setting^9^. However, the chick heart is exceptionally accessible during development and displays high molecular, anatomical, and electrophysiological homology to the hearts of mammals^10–14^. Therefore, we sought to optimize and validate a low cost, tractable, methodology to stably introduce exogenous DNA constructs into the developing chick heart. Here we present a simple, cationic lipid-based transfection system and a toolkit of integrating DNA plasmids that can be used to rapidly create genetically mosaic hearts ideal for high resolution imaging and single cell analysis. This system has several advantages over current technologies including: 1) cellular perturbations can be conducted without globally altering cardiac function, meaning downstream effects can be analyzed under normal hemodynamic conditions; 2) genetically manipulated cells can be compared with control cells within the same heart eliminating many sources of experimental variability (stage, sex, strain, etc.); 3) multiple genetic manipulations can be performed in the same cell *in vivo*; 4) large numbers of manipulated cells can be isolated from a single heart, 5) genetically encoded biosensors can be employed for real-time/longitudinal studies of physiological maturation; and 6) multiple fluorescent molecules can be targeted to subcellular locales in tandem for live-imaging of cytoarchitectural development. As such, we have identified a simple but powerful platform for examining cardiac development that combines physiological relevance of transgenic models with the flexibility of cell culture techniques.

## Results

### Cationic lipids can be used to rapidly and specifically introduce exogenous DNA into the developing heart

Viral-mediated transduction, electroporation, and chemical transfection have all previously been used to introduce DNA constructs into avian embryonic tissue^15–21^. While generally effective, each method has significant limitations. DNA delivery through infection requires the generation of viral particles which is time-consuming, relatively expensive, and has limitations in the size of the cargo DNA that can be packaged^16^. Electroporation requires a physical cavity to inject DNA, space to place electrodes, and a high degree of optimization (voltage, pulse number, pulse duration, etc.)^15–17^. Chemical transfection can be difficult to target and there is a high degree of variability in efficacy among different cell types. However, given its relative ease, low cost, and flexibility, we focused on chemical transfection as a potential approach to develop a protocol for *in vivo* cardiac somatic transgenesis.

**Figure 1:**
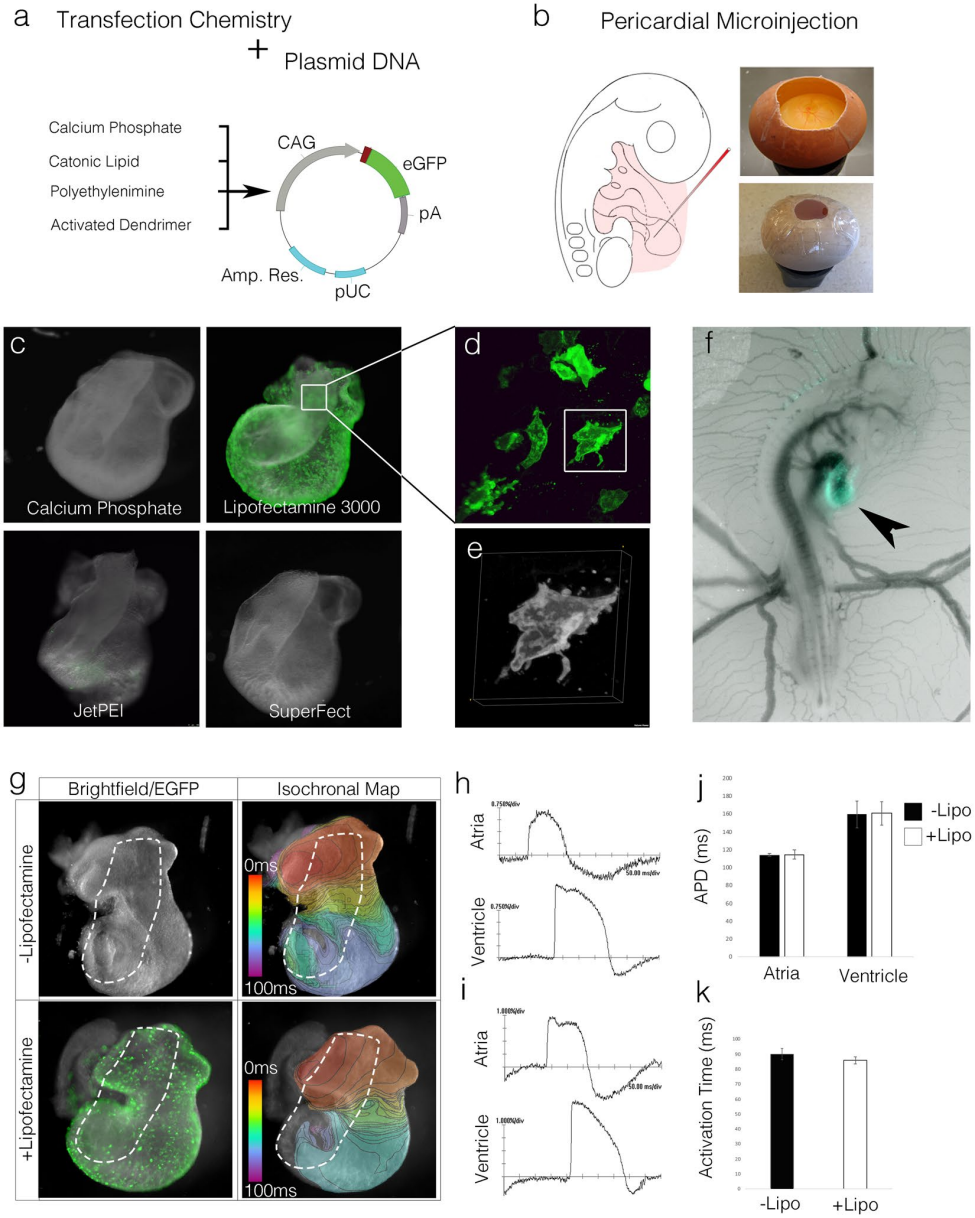
Chemical transfection of the embryonic heart. (**A**) Diagram of reagents tested for *in vivo* cardiac transfection. (**B**) Reagents were microinjected into the pericardial space of windowed HH16 embryos, eggs were then sealed and incubated to desired stages. (**C**) Representative images of hearts isolated 16 hrs. post transfection with each of the tested reagents. (**D**) Higher magnification image of an atrial myocyte expressing the palmEGFP 16 hrs. post transfection with Lipofectamine 3000. (**E**) Volumetric reconstruction of the cell indicated in (**D**). (**F**) *in ovo* image of an embryo 16 hrs. post transfection demonstrating that pericardial injection of Lipofectamine 3000/plasmid DNA reagent preferentially targets the heart (black arrowhead). (**G**) Comparison of electrical activity in control vs Lipofectamine 3000 transfected hearts. Isochronal maps are drawn at 2 ms/div. Outflow tracts (white dashed lines) were removed prior to imaging. (**H**) Atrial and ventricular wave traces (dF/F0) from untransfected heart. (**I**) Atrial and ventricular wave traces (dF/F0) from heart transfected with Lipofectamine 3000. (**J**) Comparison of Action Potential Duration (APD) between control and transfected hearts (n = 6 per condition). (**K**) Comparison of total activation time (Ventricular dV/dT max − Atrial dV/dT max) between control and transfected hearts (n = 6 per condition).

Thus, we screened a variety of transfection chemistries for their effectiveness at delivering DNA plasmids into the developing heart. Based on previous studies^20,21^, these included calcium phosphate, branched dendrimers (SuperFect), cationic polymers (JetPEI), and cationic liposomes (Lipofectamine). Initially, plasmid DNA containing the synthetic CAG promoter (CMV enhancer, chicken b-actin intron, rabbit beta globin splice acceptor)^22^ driving a palmitoylated membrane targeted EGFP (CAG-palmEGFP) was mixed with each of these transfection reagents and microinjected into the pericardial space surrounding the hearts of Hamburger Hamilton stage 16 (HH16)^23^ embryos (Fig. 1A,B). Hearts were then examined for EGFP expression following 16 hrs. of incubation. By far, Lipofectamine 3000 displayed the highest *in vivo* transfection efficiency, resulting in rapid and robust expression of EGFP in all regions of the heart (Fig. 1C). Of note, microinjection of the DNA plasmid/Lipofectamine reagent into the pericardial space resulted in highly specific expression with no transfected cells detected in the remainder of the embryo proper and only a few EGFP positive cells present in the extra embryonic vasculature and chorionic membrane. These data demonstrate that a transfection protocol based on Lipofectamine can be used to rapidly and specifically transfect embryonic cardiac cells *in vivo*.

Mechanistically, Lipofectamine relies on positively charged cationic lipids to complex and compact negatively charged plasmid DNA, packaging exogenous genetic material so it can be delivered into the cytoplasm of the target cells^24,25^. To rule out the possibility that high local concentrations of charged lipoplexes could influence the long-term excitability and/or electrical activity of transfected hearts, we examined action potential characteristics, conduction velocity, and overall activation patterns in hearts exposed to Lipofectamine. As above, hearts were transfected at HH16 and incubated for 16 hrs. Cardiac tissue was then isolated, stained with the voltage sensitive dye, Di-4-annepps, and live imaged at 2000 frames per second^13,26^. Importantly, action potential characteristics were similar between un-transfected and transfected atrial and ventricular myocytes with no detectable differences in conduction pattern, upstroke velocity, or action potential duration (Fig. 1G–J). Furthermore, total activation time required to depolarize the heart was unchanged between transfected and untransfected hearts demonstrating that conduction velocity was unaffected by Lipofectamine treatment (Fig. 1K). Collectively, these data demonstrate that Lipofectamine is an effective means to transfect embryonic cardiac tissue *in vivo* and that treatment with Lipofectamine has no detectable adverse effects on the electrical activity of the heart.

### Plasmids containing transposable elements are maintained in embryonic cardiac tissue throughout morphogenesis

**Figure 2:**
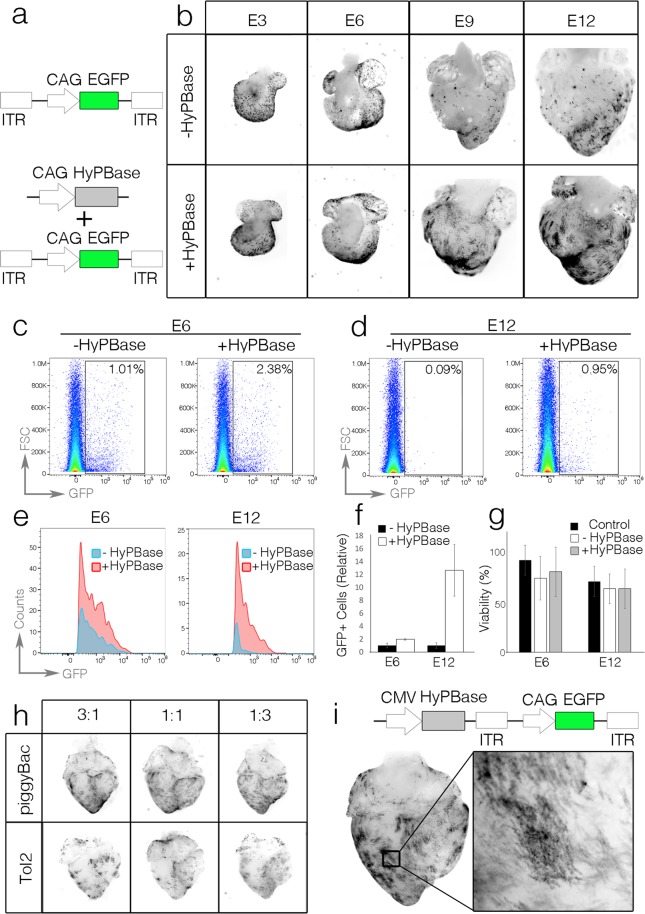
Stable expression of exogenous expression cassettes. (**A**) Diagram of expression cassettes tested. (**B**) Time series of GFP expression (black) in hearts transfected with or without HyPBase enzyme. Images were grayscaled and inverted. (**C**) Flow cytometry data from dissociated E6 hearts (88 hrs. post transfection). Plots are presented as Forward Scatter vs GFP intensity. (**D**) As in (**C**), for hearts dissociated at E12 (240 hrs. post transfection). (**E**) Quantification of cell counts vs GFP intensity from (**C**) and (**D**). Note: both the number of GFP positive cells and the intensity of GFP drops in E12 hearts that were not transfected with HyPBase. (**F**) Relative number of GFP positive cells with and without HyPBase cotransfection (n = 3 hearts per group). (**G**) Percent viability among windowed control embryos, embryos transfected without HyPBase and embryos transfected with HyPBase. (**H**) Comparison of E9 hearts transfected with different ratios of transposase to transposable element using both the PiggyBac and Tol2 transposase systems. (**I**) Heart transfected with a single plasmid containing both a HyPBase and integrating EGFP expression cassette.

Unlike viral-mediated transduction, genomic integration of chemically transfected plasmid DNA is rare. Therefore, it would be predicted that cell division in highly proliferative tissue, such as the developing heart, would dilute plasmid concentrations and inhibit high levels of ectopic expression over prolonged time-periods^27–29^. Therefore, to determine whether long-term transfection could be achieved in the developing heart, we tested a plasmid containing CAG-palmEGFP flanked by inverted terminal repeat sequences (ITRs) recognized by the piggyBac transposase^20,30–32^ (Fig. 2A). When coexpressed with the piggyBac enzyme, this EGFP expression cassette should integrate into the host cell genome.

The transposable palmEGFP construct was injected into the pericardial space of HH16 embryos either with or without an accompanying plasmid containing the hyperactive version of the piggyBac transposase (HyPBase)^33^ (Fig. 2A) and embryos were then incubated for an additional 16 hrs. (E3, HH18–19), 88 hrs. (E6, HH28–29), 160 hrs. (E9, HH35), or 240 hrs. (E12) (Fig. 2B). As expected, no obvious differences in transfection efficiency were noted between HyPBase negative and HyPBase positive transfections following 16 hrs. of incubation (Fig. 2B). However, over the next several days, the relative number of transfected cells per heart in HyPBase negative transfections decreased dramatically in comparison to HyPBase positive transfections (Fig. 2B). To quantify this, flow cytometry was used to determine the percentage of palmEGFP positive cells with or without HyPBase. At E6 (88 hrs. post transfection), the percentage of palmEGFP positive cells was 1.96-fold higher with HyPBase than without. By E12 (240 hrs. post transfection), the number of palmEGFP cells in the HyPBase positive hearts was 12.67-fold higher than the HyPBase negative embryos (Fig. 2C,D,F). The relative intensity of EGFP was also much more stable in hearts that were cotransfected with HyPBase than those without (Fig. 2E). Importantly, no significant difference in embryonic viability was noted between control (untransfected), HyPBase negative, and HyPBase positive transfected embryos suggesting that piggyBac-mediated plasmid integration was not inducing embryonic lethality (Fig. 2G). These data demonstrate that long-term, stable, somatic transgenesis can be achieved in the developing heart using transposable DNA plasmids.

It has previously been demonstrated that the ratio of transposase enzyme to transposable DNA can impact the efficiency of genomic integration^34^. To test whether the relative levels of transposase greatly altered transgene detection in our system, we varied the ratio of transposase encoding plasmid to transposon encoding plasmid over a 9-fold range using two separate transposable systems, piggyBac and Tol2^34,35^. In our hands piggyBac was the more efficient expression system in the heart, and the percentage of transgene positive cells at E12 did not dramatically differ across the ratios tested (1:3, 1:1, 3:1) (Fig. 2H). Importantly, these data indicated that a ratio of approximately 1:1 (transposase: transposable element) was effective for long-term expression. Therefore, we generated a single plasmid containing piggyBac ITR’s which contained two expression cassettes: the CMV promoter driving HyPBase and the CAG promoter driving EGFP (Fig. 2I). Using this plasmid backbone, stable expression was readily achieved without the need for cotransfection (Fig. 2I).

### Cell-type specific promoters can target expression to cardiomyocytes

**Figure 3:**
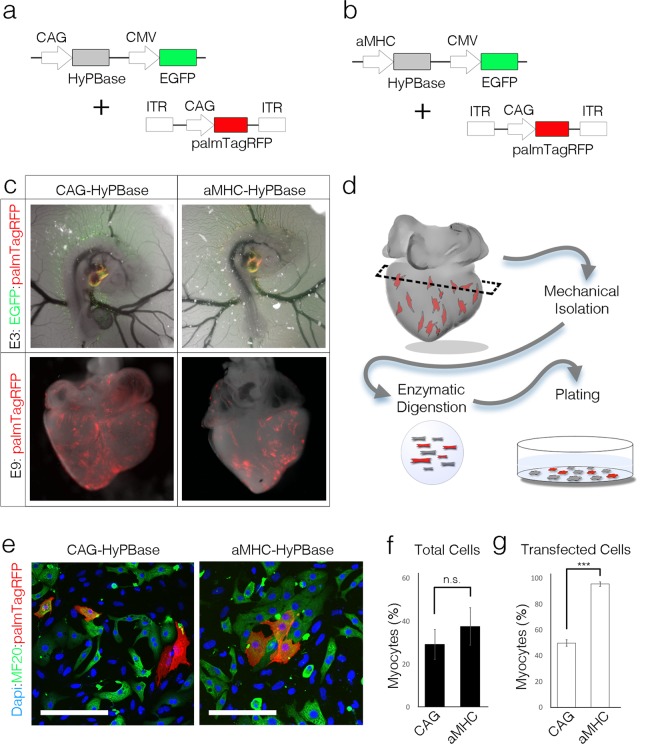
Promoter-based cell-type targeting. (**A**) Diagram of cotransfection using the ubiquitous CAG promoter to drive HyPBase and integrating palmTagRFP reporter. (**B**) As in (**A**), using the mouse aMHC promoter to drive HyPBase. (**C**) Comparison of plasmid expression 16 hrs. and 160 hrs. post transfection. (**D**) Diagram of dissociation protocol. (**E**) Comparison of MF20+ (green), palmTagRFP+ cells between CAG-HyPBase and aMHC-HyPBase transfected cells, asterisks indicate non-myocytes (MF20-). Scalebar = 100 um (**F**) Quantification of the percentage of myocytes following dissociation and culture of CAG-HyPBase and aMHC-HyPBase transfected hearts (n = 5547 cells, 3 biological replicates per group). (**G**) Quantification of the percentage of palmTagRFP positive cells that were myocytes following dissociation and culture of CAG-HyPBase and aMHC-HyPBase transfected hearts (n = 387 cells, 3 biological replicates per group).

The heart consists of a variety of myocardial and non-myocardial sub-lineages. We therefore examined whether our transfection protocol could be used to target a specific cell type in the heart by altering the promoter sequences used to drive expression. We tested two promoter sequences, chick Cardiac Troponin (cTNT) and the mouse alpha-myosin Heavy Chain (aMHC), that have previously been used to achieve muscle expression in other model organisms^36–38^. Both the cTNT and aMHC promoter sequences drove expression in the heart when cloned upstream of fluorescent reporter genes, though levels were lower than those observed using either of the ubiquitous CAG or CMV promoters (data not shown). To improve detection efficiency and to test cell type specificity, the cTNT and aMHC promoters were cloned upstream of HyPBase in an effort to restrict integration of a reporter construct (ITR-CAG-palmTagRFP-ITR) to myocytes. Both promoters were placed into a non-integrating plasmid (which contained a CMV driven EGFP reporter) and their ability to restrict the palmTagRFP reporter construct was compared with a CAG driven HyPBase (Fig. 3A,B, SFigure 1). 16 hrs. post transfection, the HyPBase plasmids (EGFP+) and the reporter plasmids (palmTagRFP+) could be detected in all hearts examined. Following 160 hrs. of incubation, however, very few EGFP positive cells could be detected while patches of palmTagRFP positive cells were maintained under all conditions, suggesting reporter construct integration had occurred (Fig. 3C, SFigure 1). To determine cell types that displayed reporter construct expression, hearts were enzymatically digested and cell suspensions were plated on fibronectin coated dishes (Fig. 3D). Cells were then stained with the muscle marker MF20 and the percentage of myocytes vs non-myocytes that were palmTagRFP positive were calculated. Using the ubiquitous CAG promoter to drive HyPBase resulted in 49.8 +/− 2.6% of palmTagRFP positive cells being myocardial. Interestingly, the cTNT driven HyPBase did not significantly enrich the fraction of transfected cells that were myocytes over CAG, suggesting that the cTNT promoter is not an effective means of achieving cardiomyocyte specific integration in this system (SFigure 1). Conversely, 95.4 +/− 1.8% of palmTagRFP transfected cells in the aMHC-HyPBase cotransfected hearts were MF20 positive, suggesting that the mouse aMHC promoter effectively restricted reporter construct expression to the desired lineage (Fig. 3E–G). Collectively these data demonstrate that promoter activity can be measured through either direct expression or selective integration of a reporter construct, and that cell types can be targeted for stable expression by altering the promoter sequence driving HyPBase.

### Direct transection allows for multiplexed cellular analysis in the same heart

**Figure 4:**
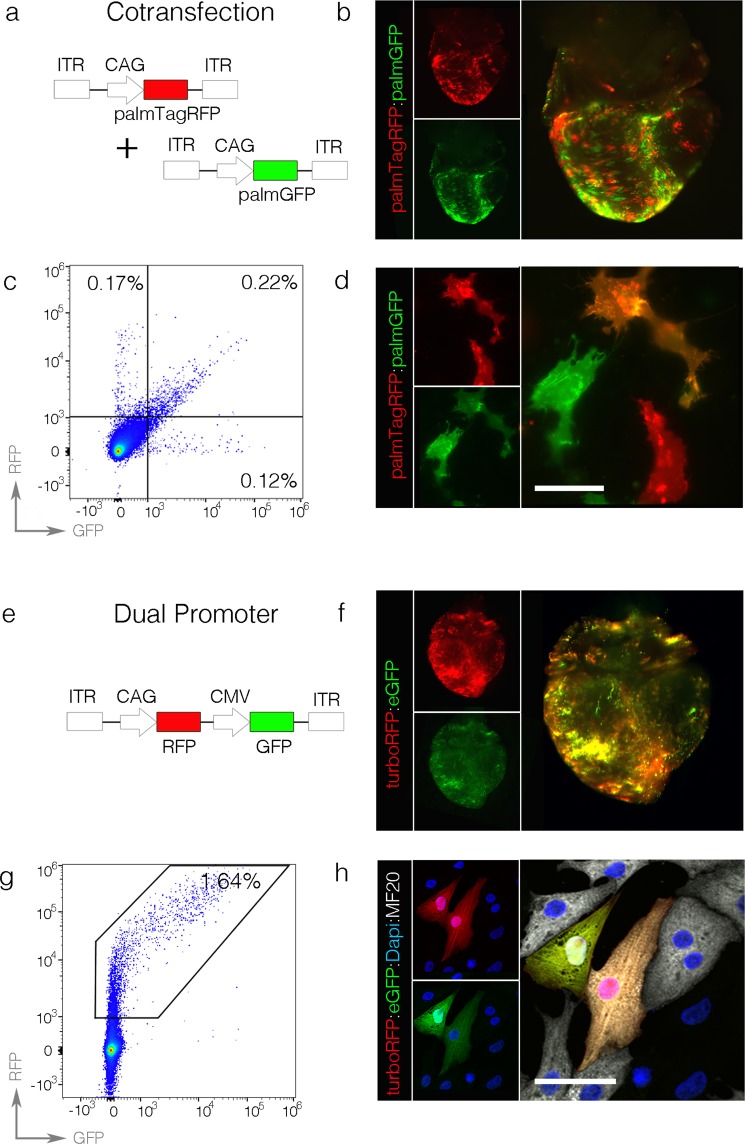
Introduction of multiple expression cassettes in the same heart. (**A**) Diagram of plasmids used in cotransfection studies. (**B**) Single channel and merged image of palmTagRFP and palmEGFP expression 160 hrs. post transfection. (**C**) Flow cytometry plot of a dissociated heart 160 hrs. post transfection with the constructs from (**A**). (**D**) Detection of palmTagRFP, PalmEGFP, and copositive cells following dissociation and culture of transfected hearts. Scalebar = 50 um. (**E**) Diagram of a dual promoter construct. (**F**) Single channel and merged images of a heart 160 hrs. post transfection. (**G**) Flow cytometry plot of a dissociated heart 160 hrs. post transfection with the construct from (**E**). (**H**) Dissociation and culture of cells from a heart transfected with the dual promoter construct demonstrating the absolute intensity of turboRFP to EGFP intensity can vary among individual cells. Scalebar = 50 um.

Creating genetic mosaics, in which cells carrying multiple modifications can be analyzed in the context of a fully functional wild-type heart, would be a powerful corollary technology to current germline transgenic systems. Furthermore, creating multiple pools of control/manipulated cells in the same heart (in which differences in stage, sex, hemodynamics, and biomechanics are automatically normalized) would allow for direct pairwise statistical analysis to be conducted between unmodified and modified cells. We therefore tested the fidelity of two approaches for introducing more than one perturbation in the same heart: cotransfection of multiple plasmids and transfection of a single plasmid containing multiple expression cassettes. For cotransfection studies, we mixed transposable constructs encoding CAG-palmTagRFP and CAG-palmEGFP with CAG-HyPBase plasmids at a ratio of 1:1:1 (Fig. 4A). As above, hearts were transfected at HH16 and incubated for 160 hrs. Under these conditions, three transfected cell populations were readily observed by fluorescent microscopy: singly transfected palmTagRFP cells, singly transfected palmEGFP cells, and cotransfected cells expressing both fluorescent proteins (Fig. 4B). Hearts were then enzymatically dissociated into single cell suspensions and analyzed via flow cytometry or placed in culture. Consistent with fluorescent microscopy, flow cytometry demonstrated that 23.8 +/− 7.1% of transfected cells were palmTagRFP positive, 19.4 +/− 3.6% were palmEGFP positive, and 57.0 +/− 9.8% were copositive (Fig. 4C), indicating that discrete populations of cells could indeed be generated in the same heart. Importantly, all three cell populations were easily identified in culture as well (Fig. 4D). These data demonstrate that a cotransfection strategy can be used to rapidly generate multiple genetically distinct cell populations in developing cardiac tissue.

To generate a dual expression system, a single construct was generated with two separate expression cassettes, CAG driving TurboRFP followed by the CMV promoter driving EGFP (Fig. 4E). As described above, hearts were transfected at HH16 and analyzed following 160 hrs. of further incubation. Interestingly CAG-TurboRFP appeared far brighter than EGFP in the resulting hearts despite the similar fluorescent properties of these two proteins (brightness of TurboRFP/EGFP = 1.24, quantum yield of TurboRFP 0.67 vs 0.70 for EGFP)^39,40^ (Fig. 4F). Flow cytometry confirmed that the CAG-TurboRFP signal was generally brighter than the CMV-EGFP in transfected cells by approximately one order of magnitude (Fig. 4G), suggesting the CAG promoter maybe significantly stronger than CMV in the heart. However, upon inspecting cells in culture a small minority of cells were detected in which EGFP was brighter than TurboRFP, suggesting that the two promoters may not display a fixed stoichiometry (Fig. 4H).

### Viral 2A sequence allows for polycistronic expression from a single promoter in the developing heart

**Figure 5:**
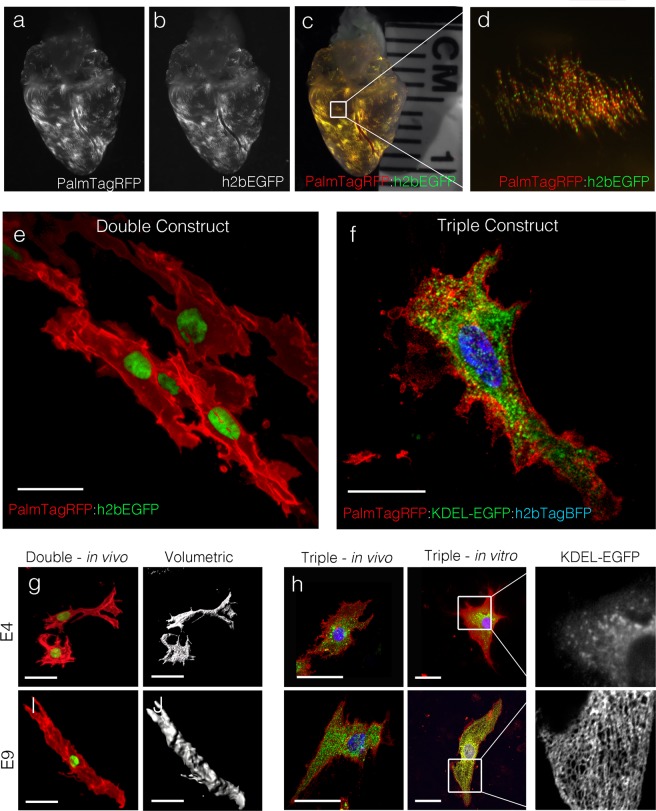
Polycistronic expression using the 2 A linker sequence. (**A**) Grayscale image of palmTagRFP expression in a heart transfected with ITR-CAG-palmTagRFP-2A-h2bEGFP-ITR. (**B**) Grayscale image of h2bEGFP expression in heart from (**A**). (**C**) Merged channels from (**A**) and (**B**). (**D**) cropped region from (**C**). (**E**) Three-dimensional reconstruction of ventricular myocytes transfected with the construct from (**A**–**D**) imaged *in situ*. Scalebar = 20 um. (**F**) Three-dimensional reconstruction of an optically bisected ventricular myocyte transfected with ITR-CAG-palmTagRFP-2A-KDEL-EGFP-2A-h2bTagBFP-ITR imaged *in situ*. Scalebar = 10 um. (**G**) Three dimensional and volumetric reconstructions of E4 and E9 atrial myocytes transfected with the construct from (**A**–**D**) imaged *in situ*. Scalebar = 20 um. (**H**) Comparison of endoplasmic reticulum/sarcoplasmic reticulum distribution in ventricular myocytes transfected with the construct from (**F**) across stages (E4–E9) and *in vivo* vs *in vitro*. Scalebar = 20 um.

Our above data demonstrates that more than one protein can be ectopically expressed in a single developing cardiomyocyte. However, under cotransfection conditions, not all cells receive both constructs and using a dual promoter system can yield unpredictable stoichiometry between transcripts. Both situations could complicate downstream interpretation and be problematic for certain experimental approaches. Therefore, we tested whether the small 2 A peptide linker sequence, which results in failed bond formation during peptide synthesis^41–43^, could be used to generate multiple proteins from a signal promoter in primary heart cells. Initially, we generated a plasmid in which the CAG promoter was used to express a single mRNA encoding membrane targeted palmTagRFP and a nuclear targeted EGFP (H2BGFP) connected by a 22 amino acid T2A linker sequence. We tested expression and maintenance of this construct by transfecting hearts at HH16 and incubating embryos for 240 hrs. This polycistronic “double construct” successfully integrated into the transfected hearts, and both palmTagRFP and H2BGFP were present in all cells examined (Fig. 5A–D). Higher magnification, confocal imaging confirmed that the TagRFP successfully trafficked to the cell membrane while EGFP was localized to the nucleus of transfected cells (Fig. 5E,G–J). These data demonstrated successful expression and separation of the two proteins encoded by the same transcript using the 2 A system. Next, we tested whether the addition of a second T2A linker could be used to generate a third peptide from a single open reading frame. Thus, we designed an additional plasmid encoding palmTagRFP, endoplasmic reticulum targeted EGFP (chicken Calrectulin signal sequence-EGFP fusion protein containing a C-terminal KDEL ER retention sequence^39^), and a nuclear targeted TagBFPII. As with the double construct, this “triple construct” expressed in the heart and the fluorescent proteins successfully trafficked to independent subcellular domains (Fig. 5F,H).

### Transfection-based biosensor introduction allows for real-time evaluation of cellular function

**Figure 6:**
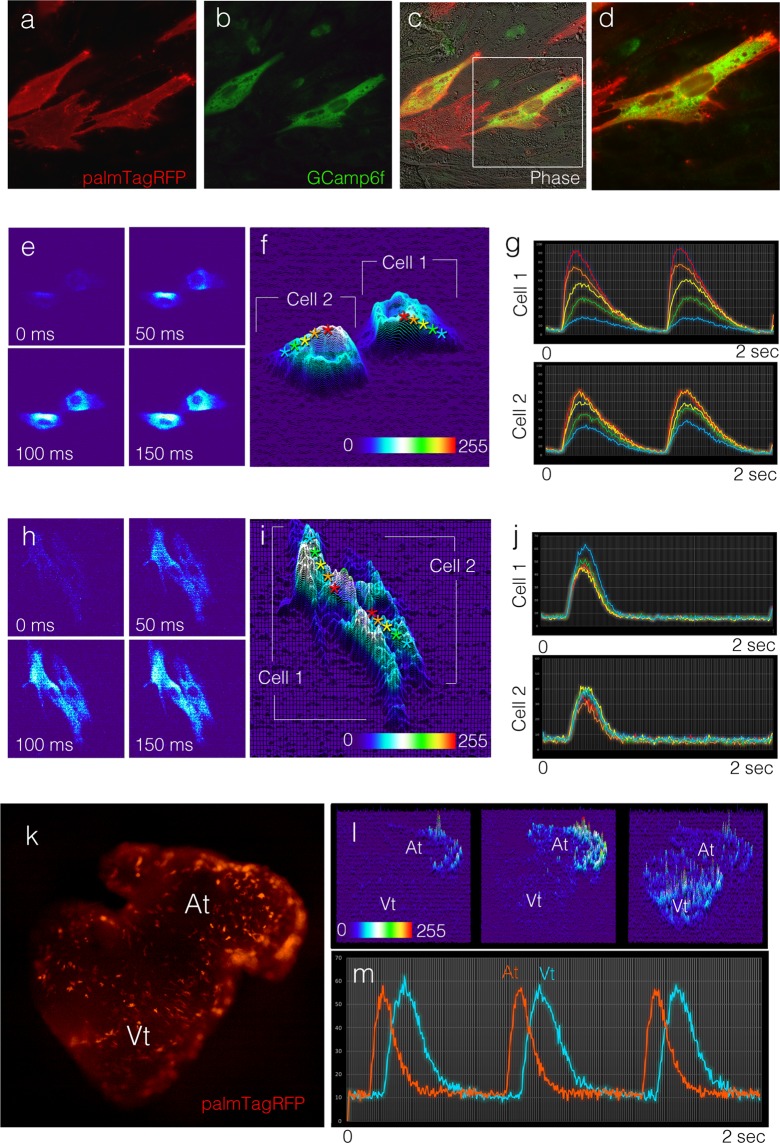
Real-time analysis of cellular function. (**A**) PalmTagRFP localization in cells isolated from a heart cotransfected with a membrane reporter and the calcium reporter GCamp6F. ((**B**) GCamp6f localization (detected with an antiGFP antibody) in cells from (A). (**C**) Images from (**A**) and **B**) overlaid on a phase contrast image. (**D**) Higher magnification image of cell from (**A**-**C**) demonstrating GCamp6f protein distribution in transfected cell. (**E**) Time series of GCamp6f intensity in a pair of transfected cells isolated from an E4 heart. (**F**) Plot of calcium transient intensity at point of maximal activity in cells from (**E**). **G**) Calcium transient traces (dF/F0) measured from areas denoted by asterisks from (**F**). (**H**-**I**) As in (**E**-**G**), for cells isolated from an E9 heart. Note both the shape of the cells and the pattern of calcium transient activation has changed. (**K**) Image of a heart cotransfected with palmTagRFP and GCamp6F. (**L**) Time series of calcium transient intensity in the heart from (**K**) (see also Sup Movie 1). (**M**) Calcium transient traces (dF/F0) from an atrial (orange) and ventricular (blue) cell from (**K**).

Currently, examining the physiological dynamics of individual cells within the context of the entire developing heart requires highly specialized imaging and data processing pipelines and is largely restricted to the zebrafish embryo^44^. To determine whether single-cell physiology could be captured in the developing chick heart, we examined whether genetically encoded biosensors could be introduced into developing cardiomyocytes via our transfection technique. We sub-cloned the calcium-indicating protein, Gcamp6F^45^, into our integrating DNA plasmid backbone and cotransfected this construct with our membrane targeted palmTagRFP plasmid. This approach was designed to allow us to simultaneously view the borders of transfected cells as well as quantify internal calcium dynamics. Initially, we plated cells from transfected hearts and examined GCamp6F protein expression and distribution using an anti-GFP antibody that recognized GCamp. Consistent with previous results, we detected three populations of transfected cells in culture, palmTagRFP positive cells, GCamp6F positive cells, and copositive cells. Using the membrane targeted palmTagRFP to define the cell borders, we confirmed that GCamp6F was expressed and distributed throughout the cytoplasm of transfected cells (Fig. 6A–D). We next examined Gcamp6f activity in plated cells isolated from hearts 16–24 hrs. post transfection (E3-E4) and 120 hrs. post transfection (E9). Interestingly, cells plated from younger hearts display calcium transients that initiated in perinuclear regions and propagated slowly towards the cell periphery (Fig. 6E–G). Furthermore, as the calcium transient approached the cell periphery the amplitude of the signal dropped substantially (n = 25 cells) (Fig. 6F,G). In contrast, transfected cells isolated from E9 hearts displayed calcium transients that activated uniformly across the whole cell and no major differences in amplitude were noted between perinuclear domains and cell periphery (n = 32 cells) (Fig. 6H–J). These data demonstrate that the biosensor Gcamp6f can be introduced into primary myocytes via our expression system and that calcium transient maturation can be traced at subcellular resolution using this technique.

Finally, we examined GCamp6f activation in whole heart preparations. Consistent with our earlier findings, expression of GCamp6f and calcium transients could be detected in all regions of transfected hearts (Fig. 6K,L, Sup Movie 1). Importantly, calcium transient behavior could be recorded from individual cells within the heart (Fig. 6M) demonstrating that biosensor introduction via direct *in vivo* transfection can be used for real-time evaluation of cardiomyocyte physiological activity within the native electromechanical microenvironment of the heart.

### Mosaic transfection can be used to rapidly perform single cell over-expression and knockdown studies in the developing heart

**Figure 7:**
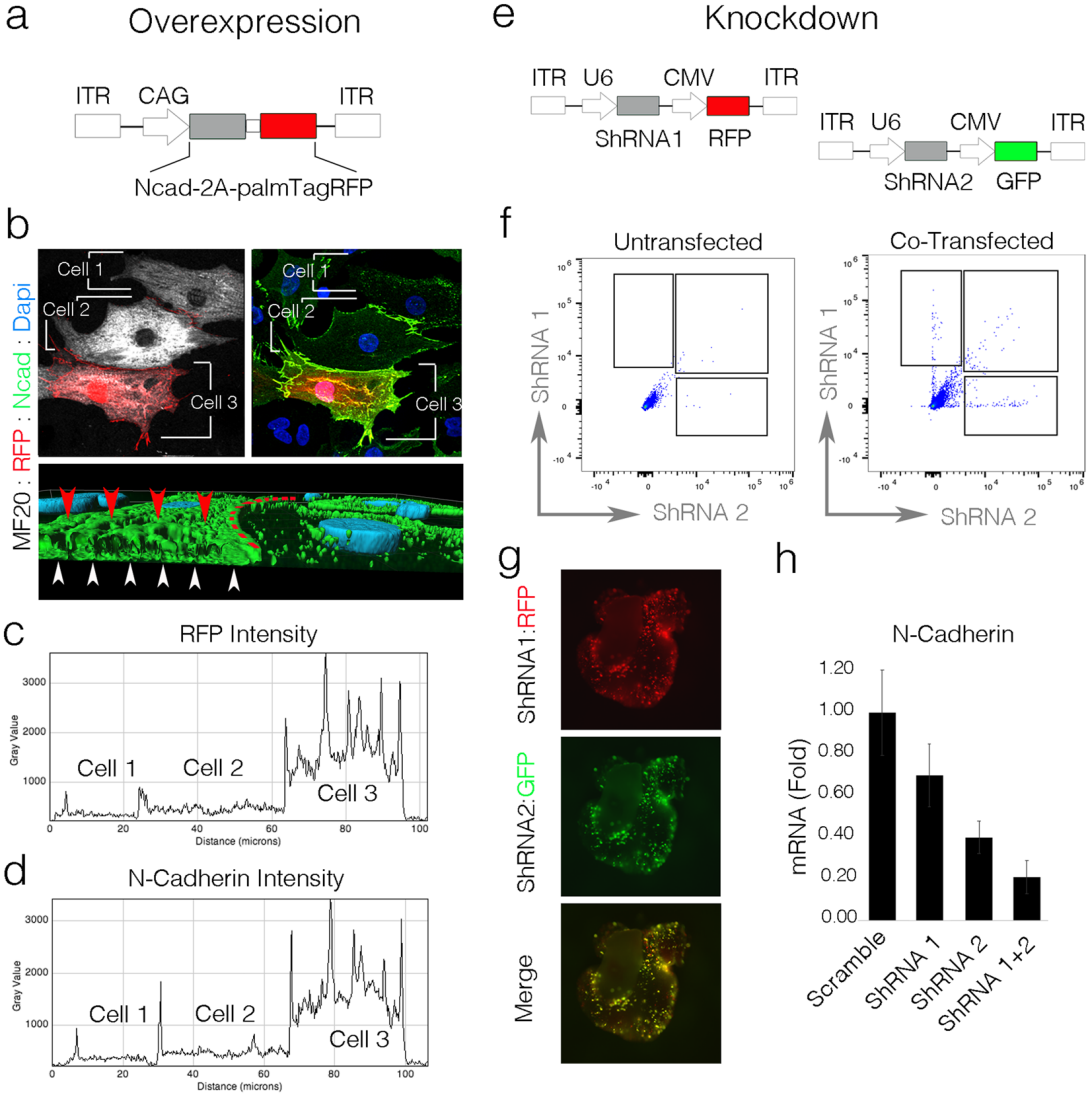
Mosaic gain-of-function and loss-of-function. (**A**) Expression cassette for N-Cadherin (Ncad) overexpression. (**B**) Confocal imaging of myocyte overexpressing Ncad and palmTagRFP. Following volumetric reconstruction, Cells 2 and 3 were optically bisected and viewed from the Z,Y orientation to demonstrate that overexpressed Ncad traffics to cell-cell junctions (red dashed line), the cell surface facing the culture media (red arrowheads), and the cell surface facing the culture substratum (white arrowheads). (**C**) Line scan of palmTagRFP intensity in cells from (**B**) (dashed line). (**D**) Line scan of Ncad immunofluorescence intensity from cells in (**B**) (dashed line). (**E**) shRNA plasmid structure for Ncad Knockdown. (**F**) Cell sorting plot from untransfected and heart cotransfected with shRNA constructs from (**E**). (**G**) Fluorescent images of cotransfected hearts showing RFP, GFP, and merged reporter channels. (**H**) qPCR measure of expression levels of Ncad mRNA in cells sorted from transfected hearts.

We next examined whether single cell gain-of-function and loss-of-function studies could be conducted in developing heart cells. To test this, we chose to modulate the expression of the adherens junction protein N-Cadherin. N-Cadherin was chosen as it has a highly stereotyped distribution in cardiomyocytes (localizing at cell-cell contacts)^46–49^ and can be detected using a well validated antibody. To generate an overexpression construct, the coding sequence for chick N-cadherin was cloned upstream of a T2A linker sequence followed by membrane targeted palmTagRFP (Fig. 7A). This construct was transfected into the heart at HH16 and embryos were incubated for 160 hrs. To confirm overexpression, hearts were dissociated, cells were plated, and immunostaining for N-Cadherin and MF20 was conducted. In untransfected control cells, N-cadherin was primarily localized to regions of cell-cell contact with punctate staining detectable along stretches of membrane not in direct apposition to another myocyte (Fig. 7B–D). In contrast, palmTagRFP positive cells displayed robust N-cadherin immunoreactivity across the entire cell body. Three dimensional reconstructions of these cells demonstrated that N-Cadherin was detectable along all of the palmTagRFP positive cell surface including the membrane facing the culture substrate and media (Fig. 7B), indicating that the overexpressed protein was successfully being trafficked to the cell surface.

In conjunction with these overexpression studies, we also generated multiple shRNAs targeting N-cadherin. DNA constructs containing a U6 promoter and shRNA followed by a separate CMV promoter driving a reporter gene (turboRFP or EGFP) were subcloned into integrating plasmid backbones (Fig. 7E). Pairs of shRNA constructs containing different reporters were then transfected into hearts at HH16 and incubated for 72 hrs. For validation, hearts were isolated and dissociated. Fluorescent Activated Cell Sorting (FACs) was then used to capture shRNA positive cells (based on RFP and GFP expression) (Fig. 7F). Quantification of mRNA from sorted cells revealed that singly transfected cells typically demonstrated knockdown in the range of 40–60% relative to scrambled shRNA controls. However, cells co-positive for multiple shRNAs displayed knockdown levels of greater than 80% (Fig. 7H). These data demonstrate that not only can knockdown be conducted in the heart through transfection-mediated shRNA introduction, but that levels of knockdown can be titrated based on the combinations of shRNAs used and that cells with high levels of knockdown, intermediate knockdown, and no knockdown at all can be examined in the same heart. Collectively, these studies demonstrate that classic gain and loss-of-function studies can be easily performed using direct *in vivo* transfection, highlighting this technique as a tractable and rapid system to study gene function, at cellular resolution, within a fully functional developing heart.

## Discussion

In this report we describe the development of a toolkit that allows for genetic manipulations to be made directly within a wildtype four-chambered embryonic heart. Importantly, this system requires no animal husbandry or maintenance of transgenic lines. Furthermore, given that the system utilizes chemical-based delivery of exogenous DNA, alterations in experimental design can be rapidly accommodated, requiring only standard molecular cloning techniques to change the genes, networks, and/or pathways being investigated. Collectively, these features greatly reduce the time and cost needed to conduct studies; meaning that experimental pipelines can be designed, executed, and iterated in a matter of days to weeks as opposed to the months it takes to generate transgenic animal lines.

We focused on the chick embryo as a foundational system for this technique. The avian embryo has long served as a classic model of four-chambered heart development due to its large size, accessibility to manual manipulation, and its amenability to physiological recording^15,50,51^. From the standpoint of genetic manipulation however, the avian heart has been difficult to work with. Viral-mediated somatic transgenesis has been used for gain-of-function and loss-of-function studies in the chick embryo (including the heart) for more than 30 years. While powerful, viral transduction has several limitations that greatly diminish its utility. Namely, the manufacturing of virus is time-consuming and requires significant practical experience^16,18^. The level of infection can be difficult to control and the number of modified cells per embryo is often low, making downstream analysis of features such as expression levels impractical to quantify. Finally, due to limitations in the amount of DNA that can be packaged into a viral capsid, only relatively simple genetic constructs can be introduced this way^16,18^. To circumvent these obstacles, many researchers have shifted to electroporation as a means of altering gene expression in the chick. However, electroporation requires a cavity to deposit DNA and space to physically place electrodes in order to target the region of interest. The heart does not meet either of these criteria as contraction rapidly displaces injected DNA solutions and the position of the heart within the pericardial cavity does not allow for effective electrode placement. Furthermore, electroporation necessitates significant stage and tissue-dependent optimization relating to the voltage applied, number or pulses, and pulse duration to ensure the viability of transduced cells. Collectively, these limitations highlight that current techniques are not well-suited for somatic transgenesis in the developing heart.

Previous work has indicated that a chemical-based transfection can be used to introduced plasmid DNA into the chick embryo. While promising, these studies had relatively low efficiency, were not able to specifically target the heart^20^, or could only be used for short-term analysis (>24 hrs.)^21^. Despite these previous limitations, the ease-of-use and the flexibility available through chemical transfection, led us to examine whether a standard protocol for *in vivo* cardiac transfection could be generated and optimized. Our data demonstrate that the combination of lipofectamine-based chemistry and integrating DNA plasmids can be used for a large variety of genetic manipulation, which opens the way toward a host of novel experimental design opportunities that can now be explored in the heart.

It should be emphasized, however, that there are several potential limitations that should be considered in relation to this technique. While piggyBac mediated genomic integration has been shown as an effective means for stable transgenesis in a variety of cell lines and animal models, including chick^20,30,53–56^, the current report has not followed transfected hearts out through adulthood or demonstrated continuity of plasmid DNA with the host chromosomal DNA. Our analysis demonstrates that stable, long-term, expression in the developing heart is markedly improved using the piggyBac system, but we cannot rule out that expression constructs may be lost over much longer time-scales. Furthermore, piggyBac has been shown to meditate integration broadly across a host genome^57,58^ and the number of integrations per cell can vary depending, in part, on the ratio of transposable element to transposase enzyme^34,53,57,59^. While we observed no changes in cardiac function or obvious drops in either embryonic or cellular viability using lipofectamine and/or the piggyBac transposase system, the potential for non-specific alterations to the genome does necessitate strong control studies. Given the simple nature through which exogenous DNA can be introduced into the heart using this technique, it is our expectation that more targeted approaches to genetic modification (such as CRISPR/cas9) can now also be easily applied to the developing chick heart.

In conclusion, biomedical researchers across multiple disciplines are facing challenges associated with how to condense, organize, and assign functional significance to the vast amounts of genomic and proteomic data that are now routinely being generated. The next generation of medical breakthroughs will rely on the mechanistic insight attained via these large data sets to design approaches to combat human disease. By extension, we will need experimental modalities that allow for creative application of data derived from current profiling studies to predictively test how cell biological behaviors are influenced. We have, therefore, designed and validated a simple *in vivo*, cardiac bioengineering platform in which the activity of single cells can be analyzed in the context of a fully functional organ. The speed, low cost, and ease of this system means that hypotheses can be rapidly tested and the decision to follow up or disregard experimental approaches can be made without major outlays of resources on the part of investigators. It is our expectation that the platform can be used to address a variety of novel discovery and design-based applications in the heart, allowing cardiac research to fully exploit the breadth of information being acquired through current “omic” technologies.

## Materials and Methods

### Embryo Handling

White Leghorn Horn Chicken eggs were obtained from Pilgrim Pride Hatchery (Siler City, NC, USA) and incubated in a humidified incubator at 38 °C. Stages were determined based on criteria established by Hamburger-Hamilton^23^. All animal procedures were approved by our American Association for Accreditation of Laboratory Animal Care committee and all experiments were performed in accordance with relevant guidelines and regulations.

### Windowing Eggs

Eggs were placed horizontally and sterilized with 70% isopropanol alcohol. Angled forceps were used to puncture the flat end of the egg shell, making a hole approximately 1 mm in diameter. An 18 G needle was used to remove approximately 5 ml of albumin. Transparent tape was applied to the top of the shell. After scoring with angled forceps, a circular hole was cut in the top of shell. The embryo was then covered with warm Hanks Balanced Salt Solution (HBSS, Gibco) [1.26 mM CaCl_2_, 0.49 mM MgCl_2_–6H_2_O, 0.41 mM MgSO_4_-7H_2_O, 5.33 mM KCL, 0.44 mM KH_2_PO_4_, 4.17 mM NaHCO_3_, 137.93 mM NaCl, 0.34 mM Na_2_HPO_4_, 5.56 mM D-Glucose] and sealed with parafilm until ready for transfection. After transfection, the eggs were sealed with transparent tape.

### Transfection

Micropipettes were prepared by pulling glass capillaries (1.0 OD/0.7, World Precision Instruments) using a micropipette puller (HEKA Instruments). Transfection reagents were prepared as follows: 1000–1500 ng of plasmid DNA was added to Opti-MEM (Gibco) to a final volume of 25 ul. In a separate tube, 2 ul of Lipofectamine 3000 (Invitrogen), Superfect (Qiagen), JetPEI (Polyplus), or CaPO_4_ (Invitrogen), was added to 23 ul of Opti-MEM. After incubating for 5 minutes at room temperature, transfection reagents were combined with plasmid DNA solution and incubated for 5 mins at 37 °C. The transfection reagent was then backloaded into the pulled glass micropipettes and the pipette was mounted onto a pressure microinjector (FemtoJet, Eppendorf). Reagent was then microinjected into the pericardial space of HH16 embryos (5–10 pulses at 100–150 hectopascals). Following injection, approximately 1 ml of 1x HBSS solution was added on top of the embryo and the window was sealed with clear tape. Embryos were then placed in a humidified incubator at 38 °C and developed to target stages.

### Dissociation

Transfected embryos were isolated from windowed eggs using curved tenotomy scissors and placed into sterile pre-warmed 1X HBSS. Hearts were removed, minced and placed in a flask with fresh, pre-warmed, HBSS containing 0.17% trypsin (Sigma-Aldrich, St. Louis, MO) and 0.085% collagenase (Sigma-Aldrich). Cardiac tissue was then incubated at 37 °C, 5% CO_2_ for 25 min and the heart homogenate was then centrifugated at 3000 rpm for 5 minutes. The supernatant was removed and the pellet was washed twice with proliferation media [Dulbecco’s Modified Eagle’s Medium (DMEM, Sigma Aldrich), 15% fetal bovine serum (FBS Advantage, Atlanta Biologicals, Flowery Branch, GA), 1% Penicillin-Streptomycin (Gibco).

### Plating cells

Enzymatically dissociated cells were filtered through a 100 um nitrex filter (Olympus). Cells were plated on glass bottom culture dishes treated with fibronectin (2 ug/cm^2^) at a density of 750,000 cells per cm^2^. Cells were then incubated at 37 °C, 5% CO^2^. For immunohistochemistry, cultures were washed twice in 1X HBSS and fixed with 2% PFA for 20 minutes.

### Flow cytometry/cell sorting

Enzymatically dissociated cells were resuspended at a concentration of 3,000,000 cells/ml, filtered through a 40 um nitrex filter (Olympus), and collected in a 5 ml polypropylene tube. For live/dead stain analysis, cells were stained with DAPI (0.1 ug/ml). The cell suspensions were stored on ice prior to loading into the cell sorter.

All flow cytometry and Cell Sorting were conducted using three biological replicates per condition. For Flow Cytometry, data acquisition was done using an Attune NxT Acoustic Focusing Cytometer and the Attune Nxt Software. Cell sorting acquisition and analysis was performed on a Becton Dickinson FACSAria III using FACSDiva 8.0.1 software. Analysis was performed using Flow Jo v10.

### RNA isolation and qPCR

For quantitative analysis of shRNA knockdown, sorted cells were collected in TRIzol (Invitrogen) and RNA was extracted using the Trizol Plus purification kit (Invitrogen) yielding approximately 500 ng of total RNA per sample group. cDNA was generated using Superscript IV (Invitrogen), per manufacturers instructions. qPCR was performed on a Quantastudio 6 cylcer (Invitrogen) using iTaq 2x SYBR green master mix (Bio-Rad). N-Cadherin was amplified using the following primers (Forward primer ATGGCAAATGAAGGTGAAGC, Reverse primer CTTCAGATGGCTGCTGTCCT) and expression levels were normalized to Gapdh and *γ*-tubulin. qPCR was conducted using 3 biological replicates, and each replicate was run in triplicate.

Live imaging of voltage sensitive dyes (Optical Mapping) – Optical mapping was performed as described previously^13,26,52^. Briefly, hearts were isolated in pre-warmed sterile Tyrode’s solution [137 mM NaCl, 2.7 mM KCl, 1 mM MgCl_2_, 1.8 mM CaCl_2_, 0.2 mM Na_2_HPO_4_, 5.5 mM D-glucose, 15 mM Hepes, pH 7.4], and allowed to recover for 20–30 min. Hearts were then transferred into staining solution (Tyrodes Solution, 10 mM Hepes, 12 mM NaHCO_3_, 10 µM Di-4-ANEPPs [Invitrogen], and 1 µM (−) Blebbistatin [Sigma], pH 7.4) saturated with 95% O2 and 5% CO2, for 10 min. Both the imaging chamber and staining solution were maintained at 37.0 °C +/− 1.0 °C throughout the imaging process using a Warner CL100 in-line media temperature control unit and a SA-OLY/2-AL stage heater. Live imaging was conducted at 2000 fps using a 14 bit, 100 × 100 pixel, CMOS Camera (MiCAM Ultima, SciMedia), mounted on a vertical THT Microscope (SciMedia).

### Immunohistochemistry

Immunofluorescence was performed using previously reported protocols^26^. After fixing in PFA, cultures were washed with 1X PBS + 0.1% Tween. Cells were then blocked for 1 hr in Blocking solution (1X PBS [pH 7.4], 1% BSA, 10% goat serum, 0.1% tween-20) at room temperature. During this time, the primary antibody was diluted in Blocking solution. MF20 and N-Cadherin antibodies (Developmental Studies Hybridoma Back) were applied at dilutions of 1:1000.

### Imaging

Following isolation, all hearts were examined and photographed using a fluorescent stereo microscope (Leica M165 FC) to confirm plasmid expression. Plated cells were imaged using an Olympus FluoView FV1000 laser scanning confocal microscope. High-magnification *in vivo* imaging was performed by embedding cardiac tissue in 2% low melting temp agarose as described previously^26^. Hearts were then imaged on a Zeiss 800 laser scanning confocal microscope. Calcium imaging using GCamp6F was conducted using a Hamamatsu ORCA-Flash4.0 CMOS camera at a rate of 100 fps at room temperature. The camera was mounted either on a Lecia M165 FC stereo microscope (Whole heart) or a Zeiss Axiovert S100TV inverted microscope using a 100× (NA 1.4) oil objective.

### Image Processing

Voltage sensitive dye-based optical mapping data was processed using BV_Ana software (SciMedia). Confocal imaging data was processed using Imaris 3D/4D Image Visualization and Analysis Software (Bitplane). GCamp6f data was processed using ImageJ V2.0.0 (imagej.nih.gov).

### Plasmids

The CAG-palmGFP plasmid was a kind gift from Dr. Timothy Sanders^30^. The CAG-HyPBase-CMV-EGFP, CAG-Tol2, ITR-CAG-RFP-CMV-GFP-ITR, U6-Ncad shRNA and ITR-CAG-palmTagRFPII-2A-h2bEGFP-ITR plasmids were designed and ordered through VectorBuilder (Shenandoah, TX). ITR-CAG-palmEGFP-ITR, ITR-CAG-palmTagRFPII-ITR, ITR-CAG-palmTagRFPII-2A-h2bTagBFP-2A-KdelEGFP-ITR were constructed by cloning synthetic gBlocks (IDT, Coralville, Iowa) into the ITR-CAG-palmTagRFPII-2A-H2BEGFP-ITR plasmid using Gibson assembly cloning (GeneArt). cTNT-HyPBase-CMV-EGFP and aMHC-HyPBase-CMV-EGFP were constructed by PCR amplifying the −550 bp chick cTNT promoter^36^ and aMHC promoters^37,38^ using Platinum SuperFi PCR Master Mix (Invitrogen) and swapping them into the CAG position of the CAG-HyPBase-CMV-EGFP using Gibson cloning. ITR-Ncad-2A-tagRFPII-ITR plasmid was generated by PCR amplifying full length chick N-cadherin and palmTagRFPII and sub cloning them into the ITR-CAG-palmTagRFPII-2A-h2bEGFP-ITR plamid on either side of the 2 A sequence. CMV-HyPBase-ITR-CAG-palmEGFP-ITR was generated by PCR amplifying CMV and HyPBase from CAG-HyPBase-CMV-EGFP and using Gibson cloning to insert them into the backbone of ITR-CAG-palmEGFP-ITR. A synthetic gBlock encoding Gcamp6F was then cloned into the palmEGFP position of the resultant plasmid to generate the calcium reporter construct.

## Supplementary information

S Movie 1

Supplementary Information

## Data Availability

No datasets were generated or analyzed during the current study.
