# Supplementary material for: Simplified platform for mosaic in vivo analysis of cellular maturation in the developing heart
Source: Sci Rep. 2019 Jul 24;9:10716. doi: 10.1038/s41598-019-47009-7 (PMC6656758; doi:10.1038/s41598-019-47009-7)
Supplement: Supplementary file 2 — Supplementary Information [file 41598_2019_47009_MOESM2_ESM.pdf]

Title: Simplified platform for mosaic *in vivo* analysis of cellular maturation in the developing heart.

Authors: Julie Goudy[1,2], Trevor Henley[1,2], Hernán G. Méndez[1], & Michael Bressan[1,2,*]

Affiliations:
[1]University of North Carolina at Chapel Hill, Department of Cell Biology and Physiology.
[2]University of North Carolina at Chapel Hill, McAllister Heart Institute.

*Corresponding author: Michael_Bressan@med.unc.edu

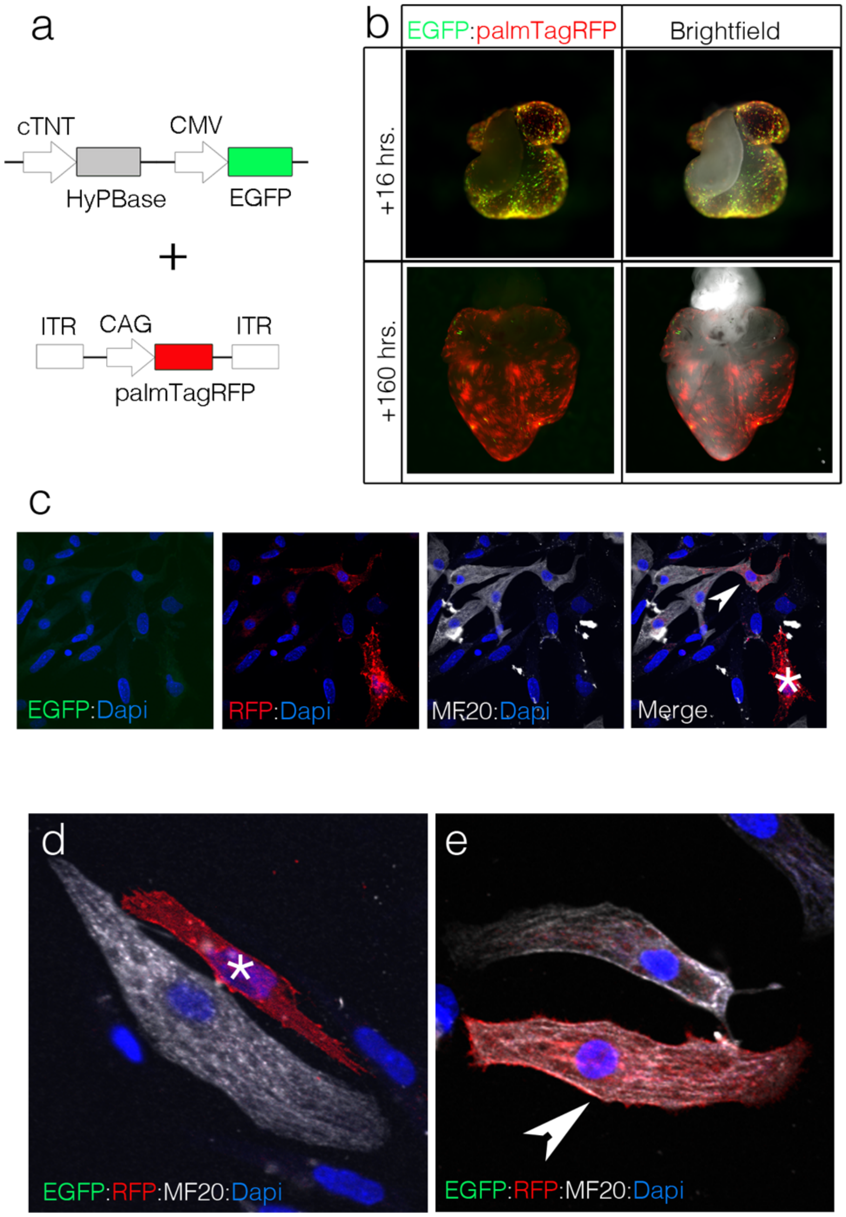

SFigure 1. Non-specific integration using the cTNT promoter. A) Diagram of plasmid combination used for transfection. **B)** Images of example hearts 16 and 160 hrs. post transfection. Note: non-integrating cTNT-HyPBase (CMV-EGFP) plasmid is clearly detectable after 16 hrs., but is largely absent following 160 hrs. **C)** EGFP, TagRFP, MF20, and merged images of cells dissociated from hearts transfected as described in (A). White arrowhead indicates MF20 positive/TagRFP positive myocyte, white asterisk indicates Mf20 negative/TagRFP positive non-myocyte. **D)** Higher magnification image of a TagRFP positive non myocyte (white asterisks). Scalebar = 20um. **E)** Higher magnification image of a TagRFP positive/MF20 positive myocyte (white arrowhead). Scalebar = 20um.

SMovie 1. Mosaic calcium transient analysis in the heart. Following cotransfection with plasmids expressing palmTagRFP and GCamp6F hearts were live imaged. Movie demonstrates single channel live imaging of palmTagRFP, GCamp6F, and a merger palmTagRFP and Gcamp6f acquisitions.
